# Plio-Pleistocene climatic change drives allopatric speciation and population divergence within the *Scrophularia incisa* complex (Scrophulariaceae) of desert and steppe subshrubs in Northwest China

**DOI:** 10.3389/fpls.2022.985372

**Published:** 2022-09-21

**Authors:** Rui-Hong Wang, Zhao-Ping Yang, Zhi-Cheng Zhang, Hans Peter Comes, Zhe-Chen Qi, Pan Li, Cheng-Xin Fu

**Affiliations:** ^1^Zhejiang Province Key Laboratory of Plant Secondary Metabolism and Regulation, College of Life Sciences and Medicine, Zhejiang Sci-Tech University, Hangzhou, China; ^2^Key Laboratory of Conservation Biology for Endangered Wildlife of the Ministry of Education, Laboratory of Systematic & Evolutionary Botany and Biodiversity, College of Life Sciences, Zhejiang University, Hangzhou, China; ^3^Key Laboratory of Biological Resources and Conservation and Application, College of Life Sciences, Tarim University, Alaer, China; ^4^Department of Biosciences, Salzburg University, Salzburg, Austria

**Keywords:** allopatric speciation, aridification, divergence, phylogeography, *Scrophularia incisa* complex

## Abstract

Numerous temperate plants and animals on the Qinghai-Tibet Plateau (QTP) are hypothesized to have differentiated due to vicariant allopatric speciation associated with the geologic uplifts. However, this hypothesis has rarely been tested through a phylogeographic study of relative species in a broader geographic context, including the QTP, Tianshan Mountains, Mongolian Plateau, and surrounding regions. To understand the speciation and diversification process of plants across this wide area, phylogeographic analysis were examined from *Scrophularia incisa* and two other closely relative species comprising *S. kiriloviana* and *S. dentata*. Thirty-two populations of the three close relatives were genotyped using chloroplast DNA fragments and nuclear microsatellite loci to assess population structure and diversity, supplemented by phylogenetic dating, ancestral area reconstructions and species distribution modelings, as well as niche identity tests. Our chloroplast DNA (cpDNA) phylogeny showed that this monophyletic group of desert and steppe semi-shrub is derived from a Middle Pliocene ancestor of the Central Asia. Lineages in Central Asia vs. China diverged through climate/tectonic-induced vicariance during Middle Pliocene. Genetic and ENM data in conjunction with niche differentiation analyses support that the divergence of *S. incisa*, *S. dentata* and *S. kiriloviana* in China lineage proceeded through allopatric speciation, might triggered by early Pleistocene climate change of increase of aridification and enlargement of deserts, while subsequent climate-induced cycles of range contractions/expansions enhanced the geographical isolation and habit fragmentation of these taxa. These findings highlight the importance of the Plio-Pleistocene climate change in shaping genetic diversity and driving speciation in temperate steppes and deserts of Northwestern China.

## Introduction

Phylogeography is a field of study concerned with principles and processes governing the geographic distributions of genealogical lineages, especially those within and among closely related species (Avise, 2000). A core objective of phylogeographic study is to recognize the responses of organisms to the past climatic oscillations, population biology and evolutionary scenarios within and between species (Abbott et al., 2000; Avise, 2009; Liu et al., 2012). In China, previous plant phylogeographic studies have broadly focused on three floristic regions or “subkingdoms” (*sensu lato*; Wu and Wu, 1996), including the Qinghai-Tibet Plateau (QTP), the “Sino-Himalayan”/Hengduan Mountain region of Southwest China, and the “Sino-Japanese” region of subtropical (Central-South-East) China and areas further north of the Yangtze River (Qiu et al., 2011; Liu et al., 2012). Together, these regions harbor the largest amount of temperate plant species diversity in the world (Myers et al., 2000; Wen et al., 2014). The arid Northwestern China is located in the interior of Eurasia at a profound distance from oceans and consequently reached by little moisture, with early uplift of the QTP, westerly winds weakened and the Mongolian-Siberian high pressure intensified, resulting in less precipitation and greater cold in this region (Fang et al., 2002a; Guo et al., 2002; Wen et al., 2016). Increased aridity with continued uplift of mountain ranges expedited the process of desertification in Northwestern China and caused large-scale expansion of deserts, such as the formation of Gurbantunggut Desert of the Junggar Basin and the Taklimakan Desert of the Tarim Basin (Sun et al., 1998; Fang et al., 2002a,b; Ding et al., 2005; Shi et al., 2005), which might have acted as an effective promoter to further adaptation of plants to various desert habitats (Wang et al., 2013; Wen et al., 2016). Even though aridification and desert formation occurred in the interior of Eurasia since the India-Asia continental collision (<ca. 50 million years age, Mya), the consequent extreme aridity and expansion of deserts in Northwest China is probably of early Miocene origin (ca. 23–16 Mya) and related to extensive uplifts of the QTP (Miao et al., 2011; Hoorn et al., 2012; Zhang et al., 2012). Moreover, some phylogeographic studies have specifically dealt with a single species of the arid flora of Northwest China, put more emphasis on the role of Quaternary climate oscillations in shaping geographical patterns of intraspecific genetic diversity and triggering glacial contractions and inter-/postglacial expansions (Li et al., 2012; Ma et al., 2012; Meng et al., 2015). However, phylogeographic study about understanding the speciation and diversification process of closely related plant taxa in this broad arid region (including the QTP, Tianshan Mountains, Mongolian Plateau, and surrounding regions) is rarely few.

**FIGURE 1:**
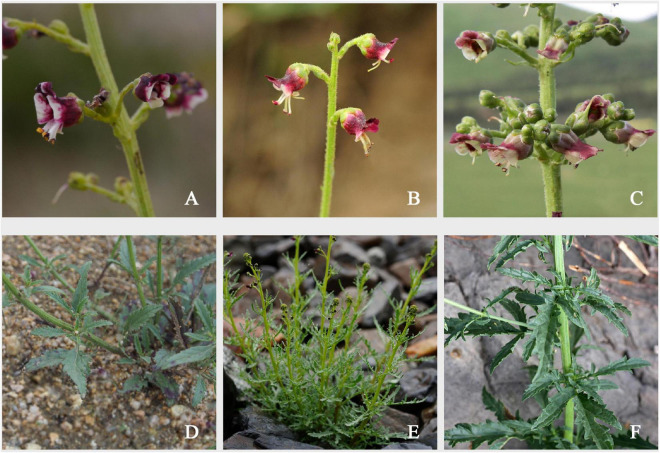
Flower and leaves morphology of the three studied species. **(A)** Flower of *S. incisa*. **(B)** Flower of *S. dentata*. **(C)** Flower of *S. kiriloviana*. **(D)** Leaves of *S. incisa*. **(E)** Leaves of *S. dentata*. **(F)** Leaves of *S. kiriloviana*. Photo A by Ye-Chun Xu; B by Jun Liu; C, F by Xue-Jing Zhan; D, E by Guo-Jun Hua.

The genus *Scrophularia* (Scrophulariaceae) comprises more than 200 species of mainly Holartic distribution in both the Old and New World (Willis, 1973; Hong, 1983; Mabberley, 1997; Hong et al., 1998). The two sections recognized (Stiefelhagen, 1910) are: sect. *Scrophualria* characterized by perennial herbs and sect. *Caninae* characterized by perennial semi-shrubs and xerophytes, as mainly distributed in Northwest China and Central Asia (Chen et al., 2014a; Wang et al., 2018). Here, we report a phylogeographic study of the “*Scrophularia incisa* complex” (sect. *Caninae*), comprising three closely related species that are mainly (but not exclusively) distributed in Northwest China and also used for medicinal purposes of treatment of exanthema and fever in Traditional Tibetan Medicine (TTM) and Mogolian Medicine (TMM), due to its secondary metabolites of the high ingredient of iridoids and phenylpropanoids (Zhang et al., 2013; Yang et al., 2014; Zhang L. Q. et al., 2014). Our preliminary analyses of nuclear ribosomal (nr) and chloroplast (cp) DNA placed this species complex as a monophyletic group embedded in sect. *Caninae* clade (Wang, 2015). Of those, *S. incisa* Weinm. presents a belt-like distribution mainly in Qinghai and Gansu Provinces as well as in Inner Mongolia of northern China, stretching westward to Central Asia and eastward to Siberia, Russia (Hong et al., 1998; Wang et al., 2014); *S. dentata* Royle ex Benth. occurs in the western and southern Tibet, northwestern India and Pakistan; and *S. kiriloviana* Schischk. is found in Xinjiang Province and Central Asia (Hong et al., 1998). All three species have very similar habitat preferences (e.g., floodplains, grasslands, and mountain valleys) and mainly differ in leaf shape and aspects of the calyx membrane (Figure 1). In detail, the leaves of *S. incisa* are toothed to lobed, rarely basally 1- or 2- segmented, with narrowly membranous margin on calyx lobes at anthesis; the leaves of *S. dentata* are lobed, deeply serrated, while the calyx lobes at anthesis have no conspicuous membranous margin, but which is obvious at the time of fruiting. Finally, in *S. kiriloviana*, the degree of leaf lobed is often apically toothed or coarsely serrate to pinnately parted, basally pinnately parted to pinnatisect, this species has a broadly membranous calyx lobe margin (Hong et al., 1998). Given the widespread distribution of the complex species (from Central Asia to Far East), we focused our phylogeographic study to medicinal species populations in Northwestern China to investigate their patterns of genetic diversity, structure and differentiation, with the overall aim to elucidate their spatiotemporal patterns of divergence and underlying causes.

Mechanisms of species diversification on the QTP and adjacent regions have attracted the attention for many years, and more than one mechanism may have played a role for a certain plant group (Qiu et al., 2011; Wen et al., 2014). Reviewing recent evidences from phylogenetic and biogeographic studies in plants, vicariant allopatric speciation associated with the geologic uplifts has been proposed as the main mechanism of species diversification on the QTP and adjacent regions (Yang et al., 2009; Xu et al., 2010). The second pattern indicates that the Tertiary and Quaternary climatic oscillations associated with the QTP uplifts have triggered and facilitated speciation and diversification, and shaped geographic genetic structure and the recolonization patterns from multiple refugia (Hewitt, 2000, 2004; Cun and Wang, 2010; Liu et al., 2012). The third pattern suggests that hybridization and introgression have contributed to the high species diversity on the QTP and adjacent areas, because of secondary sympatry during relatively stable stages between different uplifts of the QTP (Liu et al., 2006; Wang et al., 2010; Wen et al., 2014). Moreover, morphological convergence and innovations (Wang et al., 2009; Xie et al., 2011; Zhang J. Q. et al., 2014), biotic interactions (pollinator-mediated isolation, Hou et al., 2008; Niu et al., 2011; Eaton et al., 2012), and polyploidy (Stebbins, 1940, 1971; Mayrose et al., 2011) are also considered to be the important mechanisms in plant evolution.

In the present study, we used maternally inherited chloroplast DNA (cpDNA) and bi-parentally inherited nuclear microsatellite (nSSR) data, combing the (palaeo-)climatic data and distribution models, in an attempt to investigate the phylogeography and spatial-temporal process of divergence within the *S. incisa* complex. The specific aims of this study were: (1) to illuminate the phylogeographic pattern of the species complex based on plastid vs. nuclear data and species boundaries; (2) to determine the divergence times of the three focal species and intraspecific lineages as well as the underlying divergence mechanism; (3) to infer the influence of Quaternary climatic oscillations on the species complex.

## Materials and methods

### Plant materials and sampling design

In total, 522 individuals of the *S. incisa* complex were collected, including 263 individuals of *S. incisa* (11 populations from in Qinghai Province, 3 from Gansu Province, 1 from Northeastern Inner Mongolia), 232 individuals of *S. kiriloviana* (15 populations mainly distributed in Xinjiang Province), and 27 individuals of *S. dentata* (three populations from Tibet). According to our field investigations, the current population number of *S. incisa* in the Central and Western regions of Inner Mongolia is limited, possibly as a consequence of habitat loss due to over-exploitation and drought. Our sampling covers most of the distribution range of this species complex. All samples (*n* = 522) were surveyed for nSSR variation (except for the CA population of *S. kiriloviana* from West Tien-Shan in Central Asia because of its small sample size); for cpDNA, all samples of *S. dentata* (*n* = 27) were sequenced, but only subsets for *S. incisa* (*n* = 202) and *S. kiriloviana* (*n* = 177).

### DNA extraction, sequencing, and microsatellite genotyping

Total genomic DNA was extracted from leaf material that had been dried with silica-gel, using DNA Plantzol (Invitrogen, Carlsbad, CA, USA) following the manufacturer’s protocol. For the phylogeographic DNA surveys, we sequenced two intergenic spacer (IGS) regions of cpDNA (*trn*L*-trn*F and *psb*A*-trn*H). To better resolve the phylogenetic relationships among the 30 haplotypes recovered from the above two cpDNA markers, one additional cpDNA region (*trn*Q-*rps*16) was sequenced for 30 samples representative of all the haplotypes identified in the phylogeographic survey. PCR was performed using the primer sequences and amplification conditions of Scheunert and Heubl (2011, 2014, 2017). Sequences were generated with an ABI 377XL DNA-sequencer, and edited, assembled, and aligned in GENEIOUS PRO v4.8.2 (Drummond et al., 2009; available at http://www.geneious.com). All haplotype sequences identified in the present study were deposited in GenBank (see Supplementary Table 2 for accession numbers).

All DNA samples (except for population CA) were genotyped at 12 nSSR loci using primers (Scin1–12; GenBank accession numbers: JQ773338–773349) and amplification protocols developed for *S. incisa* (Wang et al., 2014; see Supplementary Table 3). PCR products were separated on a MegaBACE 1000 (CE Healthcare Biosciences, Sunnyvale, California, USA). Alleles were scored manually using GENETIC PROFILER v2.2 (GE Healthcare Biosciences).

### Phylogeographical and population genetic analyses of chloroplast DNA and nSSRs

For cpDNA, haplotypes (*h*) and nucleotide (π) diversities were estimated using DNASP v 5.1 (Librado and Rozas, 2009) at the levels of populations (*h*_*S*_, π_*S*_) and species (*h*_*T*_, π_*T*_). Networks indicating the genealogical relationships of the haplotypes were constructed in TCS v 1.21 (Clement et al., 2000) under the 95% statistical parsimony criterion. For cpDNA, we had to increase the TCS connection limit to 50 steps to link the divergent networks of the three species. Gaps (indels) detected in the cpDNA dataset were treated as single mutation events, and coded as substitutions (A or T).

The program PERMUT (Pons and Petit, 1996) was employed to compare parameters of cpDNA-based population differentiation (*N*_*ST*_ and *G*_*ST*_) of each species and/or lineage separately based on 1,000 random permutations. A mismatch distribution analysis (MDA; Slatkin and Maddison, 1989; Rogers and Harpending, 1992; Schneider and Excoffier, 1999) was conducted to examine the demographic expansions of the four major cpDNA clades identified in the phylogenetic analyses. As population structure has a limited effect on the mismatch distribution (Rogers, 1995; Bernatchez, 2001), we pooled all haplotypes of each clade and did not consider their frequencies. We used 1,000 parametric bootstrap replicates to generate an expected distribution using a model of sudden demographic expansion (Excoffier and Lischer, 2010), to calculate the sum of squared deviations (*SSD*) and raggedness index (*H*_*Rag*_) of Harpending (1994) between observed and expected mismatch distributions and to obtain 95% confidence intervals (CIs) around τ. We also calculated Tajima’s (1989) *D* and Fu’s (1997) *F*_*s*_ to assess possible expansions. The *D* and *F*_*s*_ statistics should have large negative values within a clade under the expansion hypothesis due to an excess of rare new mutations. We calculated significance of the tests with 10,000 replicates. All of these demographic tests were performed using ARLEQUIN v.3.5 (Excoffier and Lischer, 2010). When sudden expansions were detected, we used the equation τ = 2*ut* (Rogers and Harpending, 1992; Rogers, 1995) to estimate expansion times, where *t* is the expansion time in number of generations, τ is the mode of the mismatch distribution, and *u* is the mutation rate per generation for the whole analyzed sequence. We calculated *u* according to *u* = μ*kg*, where μ is the substitution rate per nucleotide site per year of the combined cpDNA regions obtained from the corresponding clock-calibrated BEAST tree (see below), *k* is the average sequence length of the DNA region under study (here, 1,310 bp), and *g* is the generation time in years (i.e., age of first reproduction); for, *g*, we assumed 3 years, according to our observation on *S. incisa* (R.H. Wang, pers.obs.).

For the nSSRs, we calculated measures of genetic diversity for each population, and across all 12 loci. The following diversity and inbreeding parameters were calculated: *A*_*R*_, allelic richness (Mousadik and Petit, 1996); *PA*_*R*_, private allelic richness; *H*_*E*_, expected heterozygosity (Nei, 1978), *H*_*O*_, observed heterozygosity; and the average inbreeding coefficient (*F*_*IS*_) across all loci. *A*_*R*_ and *PA*_*R*_ were calculated by rarifying to 12 gene copies using FSTAT and HP-RARE v1.1 (Kalinowski, 2005), respectively.

Genetic subgroups in the nSSR dataset were identified by a Bayesian analysis in STRUCTURE v2.3 (Pritchard et al., 2009) using the admixture model and assuming independent allele frequencies among populations. The number of clusters (*K*) was set to vary from 1 to 10. For each value of *K*, we performed 10 runs with a burn-in length of 10,000 and a run length of 100,000 Markov chain Monte Carlo (MCMC) replications. Two alternative methods were used to explore the true number of gene pools: by monitoring the change in average of log-likelihood of the data, log_*e*_P(*D*), of independent runs for each *K*, following Pritchard et al. (2000), and by observing the second-order rate of change of log_*e*_P(*D*) between successive *K* values (Evanno et al., 2005).

In order to quantify variation in cpDNA sequences and nSSRs among populations and clades (as identified by the TCS network and BEAST-derived tree; see below), non-hierarchical and hierarchical analyses of molecular variance (AMOVAs) were carried out in ARLEQUIN, using Φ- and *R*-statistics, respectively. The significance of fixation indices was tested using 10,000 permutations.

### Gene flow analyses

In order to test whether postglacial gene flow may obscure the genetic signatures of historical population processes, based on the nSSR dataset, we obtained pairwise estimates of postglacial gene flow (*c.* 4*Ne* generations in the past; Beerli and Felsenstein, 2001) between regional population groups (i.e., four genealogically distinct units of cpDNA) using model-based coalescent analysis in MIGRATE v3.1.3 (Beerli, 2006). This program calculates maximum likelihood (ML) estimates for mutation-scaled migration rate (*M*) [*M* = *m*μ^–1^, where μ is the mutation rate per generation (3 × 10^–3^); Udupa and Baum, 2001]. These analyses were run for three replicates under a Brownian motion mutation model with constant mutation rates for all loci and starting parameters based on *F*_*ST*_ calculations. We used uniform priors and Metropolis sampling with 10 short and five long chains with 10,000 and 100,000 sampled genealogies, respectively. Genealogies were 50 steps apart and the first 10,000 were discarded as burn-in. We used a static heating scheme at four temperatures (1, 1.5, 3, and 6) to efficiently search the genealogy space.

### Phylogenetic divergence time estimation

**FIGURE 2:**
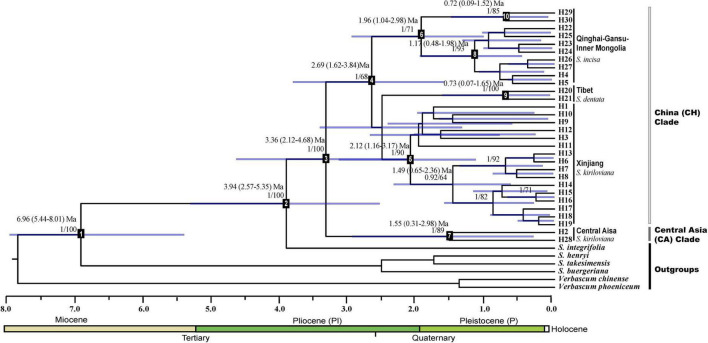
BEAST-derived chronograms of the *Scrophularia incisa* complex based on cpDNA (*trn*L–*trn*F, *psb*A–*trn*H, and *trn*Q–*rps*16). Blue bars indicate the 95% highest posterior density (HPD) credibility intervals for node ages (in Myr ago, Ma). Posterior probabilities and bootstrap values (>50%) based on maximum-likelihood (ML) analysis are sequentially labeled above nodes. Mean divergence dates and 95% HPDs for major nodes (1–10) are summarized in Table 2. Haplotypes are indicated by letter codes (H1–30).

Divergence time between cpDNA haplotype lineages was estimated under ML and Bayesian inference (BI) approaches, with six outgroup species, including *S. integrifolia* of sect. *Caninae* (a close relative according to Scheunert and Heubl, 2017), three species of sect. *Scrophularia* (*S. henryi*, *S. takesimensis*, and *S. buergeriana*), and two species of *Verbascum* (*V. chinense* and *V. phoeniceum*). Taking advantage of calibration points used in a previous phylogenetic study of Lamiales (Xu et al., 2018), we adopted the estimated median crown age of *Scrophularia* [mean, 7.88 Ma; 95% highest posterior density (HPD) interval, 2.67–14.75 Ma] to calibrate the respective root node in our tree (node 1 in Figure 2). A Yule process was specified as a tree prior. Bayesian searches were conducted in BEAST (Drummond and Rambaut, 2007) using a GTR + I + G substitution model selected by JMODELTEST (Posada, 2008) and an uncorrelated lognormal relaxed clock (Drummond et al., 2002). For the BEAST analysis, MCMC runs were performed, each of 5 × 10^7^ generations, following a burn-in of the initial 10% cycles. MCMC samples were inspected in TRACER to confirm sampling adequacy and convergence of the chains to a stationary distribution. In addition, haplotype relationships were also inferred *via* ML analysis in RAXML v7.2.8 (Stamatakis et al., 2008) under the GTR + G substitution model (as selected by JMODELTEST), and with gaps (indels) treated as missing data. Node support was assessed using 1,000 “fast bootstrap” replicates.

**TABLE 1:** The analysis of molecular variance (AMOVA) for cpDNA data and nSSR data among geographic regions and populations of *Scrophularia incisa* complex.

Source of variation	cpDNA					nSSRs				
	d.f.	Sum of squares	Variance components	Percentage of variation (%)	Φ-statistics	d.f.	Sum of squares	Variance components	Percentage of variation (%)	*R*-statistics
***S. incisa* complex**										
Among species	2	874.189	3.521 Va	57.75	Φ_CT_ = 0.58	2	23.410	0.038 Va	8.08	*R*_CT_ = 0.08
Among populations within species	29	917.278	2.418 Vb	39.65	Φ_SC_ = 0.94	539	263.468	0.054 Vb	11.50	*R*_SC_ = 0.13
Within populations	385	61.029	0.159 Vc	2.60	Φ_ST_ = 0.97	542	206.000	0.380 Vc	80.41	*R*_ST_ = 0.20
**Four geographic regions**										
Among regions	3	1098.720	4.069 Va	66.44	Φ_CT_ = 0.66	3	27.307	0.040 Va	8.51	*R*_CT_ = 0.09
Among populations within regions	28	692.747	1.897 Vb	30.97	Φ_SC_ = 0.92	538	259.571	0.051 Vb	10.86	*R*_SC_ = 0.12
Within populations	385	61.029	0.159 Vc	2.59	Φ_ST_ = 0.97	542	206.000	0.380 Vc	80.63	*R*_ST_ = 0.19
***Scrophularia incisa***										
Among populations	14	316.150	1.555 Va	94.57	Φ_ST_ = 0.95	282	131.592	0.050 Va	11.89	*R*_ST_ = 0.12
***Scrophularia kiriloviana***										
Within populations	202	39.865	0.089 Vb	5.43		283	104.000	0.368 Vb	88.11	
Among populations	13	593.869	3.698 Va	93.65	Φ_ST_ = 0.94	231	121.228	0.070 Va	15.27	*R*_ST_ = 0.15
Within populations	159	39.865	0.251 Vb	6.35		232	89.500	0.386 Vb	84.73	
***Scrophularia dentata***										
Among populations	2	7.259	0.457 Va	77.88	Φ_ST_ = 0.78	2	1.198	0.011 Va	2.58	*R*_ST_ = 0.03
Within populations	24	3.111	0.130 Vb	22.12		51	21.950	0.430 Vb	97.42	

**TABLE 2:** Summary of cpDNA-based divergence time estimation results under a Bayesian approach.

Node	Type	Calibration points (Ma)	Mean ages (95% HPD)
Node 1 (crown age of *Scrophularia*)	Secondary calibration point	6.96	5.44, 8.01
Node 2 (crown age of *Scrophularia* sect. *Tomiophyllum*)		3.94	2.57, 5.35
Node 3 (crown age of *S. incisa* complex)		3.36	2.12, 4.68
Node 4 (crown age of China clade)		2.69	1.62, 3.84
Node 5 (crown age of Xinjiang clade)		2.12	1.16, 3.17
Node 6 [crown age of Qinghai-Gansu-Inner Mongolia (QGI) clade]		1.96	1.04, 2.98
Node 7 [crown age of Central Asia (CA) clade]		1.55	0.31, 2.98
Node 8 (crown age of Qinghai-Gansu subclade)		1.17	0.48, 1.98
Node 9 (crown age of Tibet clade)		0.73	0.07, 1.65
Node 10 (crown age of Inner Mongolia subclade)		0.72	0.09, 1.52

### Ecological niche modelings and niche identity tests

Ecological niche models (ENMs) were developed for each species in MAXENT (Phillips et al., 2006) to predict suitable climate envelopes. A total of 78 collection records for *S. incisa*, 60 for *S. kiriloviana*, and 49 for *S. dentata* were obtained from the Chinese Virtual Herbarium^1^ and the National Specimen Information Infrastructure of China^2^ and Global Biodiversity Information Facility.^3^ Nineteen bioclimatic layers were downloaded from the WorldClim database^4^ (Hijmans et al., 2005) at 2.5 arc-min resolution, for current (the period 1960–1990), the LGM (c. 21,000 year before present; BP) predicted according to CCSM model and a future periods (2070) running the RCP8.5 experiment using the CCSM4 model, respectively. Highly correlated variables were identified using the “pairs” function of the R package RASTER v3.1-5 (Hijmans, 2020) and were removed to prevented potential overfitting, with five retained for analysis. These five variables with low correlation were BIO8 (mean temperature of wettest quarter), BIO10 (mean temperature of warmest quarter), BIO14 (precipitation of driest month), BIO17 (precipitation of driest quarter), BIO19 (precipitation of coldest quarter) for *S. incisa*, BIO3 (isothermality), BIO6 (min temperature of coldest month), BIO7 (temperature annual range), BIO8, BIO14 for *S. dentata*, and BIO3, BIO5 (max temperature of warmest month), BIO6, BIO11 (mean temperature of coldest quarter), BIO19 for *S. kiriloviana*. We performed 50 randomly subsampled replicate runs with 25% of observations retains for cross-validation. Models were further evaluated using Area Under the Curve (AUC) values of the Receiver Operating Characteristic (ROC) plot. AUC values greater than 0.9 indicates a very good prediction, while greater than 0.75 means the predicted model is better than a random model (Swets, 1988).

Niche identity tests were also performed to evaluate the niche similarity among the three species in ENMTools v1.4.3 (Warren et al., 2010) based on all the 19 BIOCLIM variables from the WorldClim dataset for testing the null hypothesis that *S. incisa*, *S. kiriloviana*, and *S. dentata* are occupying identical climatic environments (niches). Niche overlap was quantified using the standardized Hellinger dist enlargement in Northwesterance (*I*) and Schoener’s *D* (Schoener, 1968).

### Ancestral area reconstructions

In order to reconstruct the geographical diversification of *S. incisa* complex, the Bayesian binary MCMC (BBM) analysis implemented in RASP v3.0 (Yu et al., 2020)^5^ was performed using trees retained from the interspecific BEAST analysis (see above). To prevent biased inferences toward wide or unlikely distributions for the crown node of the ingroup, caused by uncertainties regarding the root area of the outgroup (Migliore et al., 2012; Harris et al., 2013), we pruned one outgroup for our ancestral state reconstructions. Four geographic regions representing the current distribution were defined according to floristic divisions: A, Central Asia; B, Xinjiang; C, Qinghai-Gansu and Inner Mongolia; D, Tibet (Good, 1974; Meng et al., 2015). These floristic divisions are generally based on the floral composition and vegetation types (Meng et al., 2015). The number of maximum areas at each node was set to four. To account for phylogenetic uncertainty, 5,000 out of 20,000 post-burn-in trees from the BEAST analysis were randomly chosen for BBM analysis (see also Chen et al., 2014b). We set the root distribution to null, applied 10 MCMC chains with the JC + G model running for 10^6^ generations, and sampled the posterior distribution every 100 generations.

## Results

### Chloroplast DNA diversity and population structure

**FIGURE 3:**
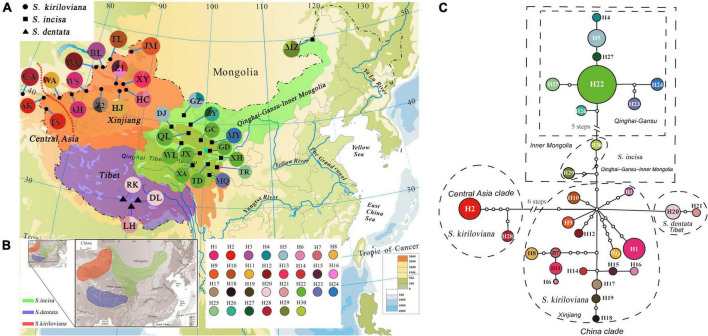
**(A)** Geographic distribution of the 30 chloroplast (cp) DNA (*trn*L-*trn*F and *psb*A-*trn*H) haplotypes (H1–H30) detected in the *Scrophularia incisa* complex (see Supplementary Table 1 for population codes). Red dashed lines denote divergences identified by TCS and phylogenetic analyses. **(B)** Geographic ranges of *S. incisa*, *S. dentata* and *S. kiriloviana*. **(C)** TCS-derived network of genealogical relationships of the 30 cpDNA haplotypes. The small open circles represent missing haplotypes. The size of circles corresponds to the haplotype frequency.

The two cpDNA-IGS regions surveyed across the 413 individuals (33 populations) of the *Scrophularia incisa* complex were aligned, with a total length of 1,310 bp and 32 substitutions and 15 indels (Supplementary Table 4). In combination, these polymorphisms identified a total of 30 haplotypes (H1–30). Of those, 1 (H28) was specific to *S. kiriloviana* in Central Asia, 16 were specific to Xinjiang (H1, H3, H6–19) and H2 was shared between Central Asia and Xinjiang. Whereas eight were specific to *S. incisa* in Qinghai and Gansu (H4, H5, H22–27), two were specific to *S. incisa* in Inner Mongolia (H29–30) and two (H20, H21) were specific to *S. dentata* in Tibet. For the species complex as a whole, the cpDNA data revealed high levels of haplotype diversity (*h*_*T*_ = 0.102) and nucleotide diversity (π_*T*_ = 0.314 × 10^–3^). On average, *S. kiriloviana* had much higher levels of within-population diversity (*h*_*S*_ = 0.132; π_*S*_ = 0.302 × 10^–3^) than *S. incisa* (*h*_*S*_ = 0.060; π_*S*_ = 0.047 × 10^–3^), whereas the three *S. dentata* populations were fixed for H20 and H21 (Supplementary Table 1 and Figures 3A,B).

The parsimony network (Figure 3B) grouped the 30 cpDNA haplotypes into two major clades (Central Asia “CA” and China) separated by seven mutational steps. Thus, each region mostly harbored a genealogically distinct set of haplotypes except Central Asia and Xinjiang sharing H2. Within the China clade, haplotypes from Qinghai-Gansu (H4, H5, H22–27), Inner Mongolia (H29–30) and Tibet (H20–21) formed interior subclades relative to those from Xinjiang (H1, H3, H6–19) (Figures 3A,B). Hierarchical AMOVAs (Table 1) apportioned 57.75% of the total cpDNA variance among the three species, with 39.65% explained by variation among populations within species; in addition, 66.44% of this total variance distributed among four geographic regions (Central Asia, Xinjiang, Qinghai-Gansu-Inner Mongolia, Tibet) with 30.97% among populations within regions. Non-hierarchical analyses revealed stronger population genetic structure in both *S. incisa* (Φ_*ST*_ = 0.95) and *S. kiriloviana* (Φ_*ST*_ = 0.94) compared to *S. dentata* (Φ_*ST*_ = 0.78) (Table 1). A significant cpDNA phylogeographic structure was indicated for the whole species complex (*N*_*ST*_ = 0.966 > *G_*ST*_* = 0.870), *S. kiriloviana* (*N*_*ST*_ = 0.915 > *G_*ST*_* = 0.843) and *S. incisa* (*N*_*ST*_ = 0.982 > *G_*ST*_* = 0.877) (all *P* < 0.05), but not for *S. dentata* (*N*_*ST*_ and *G_*ST*_* = 0.75).

### Phylogenetic haplotype relationships and molecular dating

The cpDNA tree topologies obtained from Bayesian inference (BI) (Figure 2) and maximum likelihood (ML, not shown) supported the monophyly of both the *S. incisa* complex (posterior probability, PP = 1, ML bootstrap support = 100%) with *S. integrifolia* as the sister group (PP = 1, ML = 100%) and its divisions into two well-supported main lineages: Central Asia clade (“CA”; PP = 1, ML = 89%) and China clade (PP = 1, ML = 68%). The CA clade contained haplotypes of *S. kiriloviana* from the eastern foot of the Pamirs Plateau (pops. AK, TS, WA: H2) in Southwest of Xinjiang and West Tien-Shan (pop. CA: H28; Uzbekistan) in Central Asia. Within the China clade, three lineages were resolved: (i) a strongly supported “Xinjiang” clade (PP = 1, ML = 90%) comprising all the remaining haplotypes of *S. kiriloviana*; (ii) a strongly supported “Tibet” clade (PP = 1, ML = 100%), consisting of all *S. dentata* haplotypes; (iii) a well-supported “Qinghai-Gansu–Inner Mongolia” clade (“QGI”), containing all haplotypes of *S. incisa* (PP = 1, ML = 71%), which could be further divided into Qinghai-Gansu (PP = 1, ML = 93%) and Inner Mongolia (PP = 1, ML = 85%) subclades.

Based on our BEAST-derived chronogram (Figure 2), the *S. incisa* complex likely originated in the Middle Pliocene at *c.* 3.94 Ma (node 2) and started to diversify at *c.* 3.36 Ma (node 3), whereas the crown ages of the main lineages fell into the Late Pliocene to Middle Pleistocene (China: *c.* 2.69 Ma, node 4; Xinjiang: *c.* 2.12 Ma, node 5; QGI: *c.* 1.96 Ma, node 6; CA: *c.* 1.55 Ma, node 7; Tibet: *c.* 0.73 Ma, node 9) (Table 2). For this chronogram, BEAST provided an average substitution rate of 1.07 × 10^–9^ s/s/y, which is little slower than the mean values usually reported for non-coding cpDNA regions (e.g., 1.2–1.7 × 10^–9^ s/s/y; Graur and Li, 2000).

### Demographic analyses based on chloroplast DNA sequence variation

Estimates of Tajima’s *D* and Fu’s *Fs* were generally non-significant for all cpDNA clades of the *S. incisa* complex (Table 3). By contrast, the observed mismatch distribution of haplotypes for each clade failed to reject the demographic expansion model in most cases (*SSD*, *H*_*Rag*_ values *P* > 0.01; Table 3). Nevertheless, only the mismatch distributions of the “Xinjiang” and “Qinghai-Gansu–Inner Mongolia” clades, were unimodal and general fit to the distributions expected under a demographic expansion model (Table 3 and Supplementary Figure 1). Based on the corresponding τ values, and assuming a substitution rate of 1.07 × 10^–9^ s/s/y (see above), we dated the two demographic expansions to the Early Pleistocene (Xinjiang: *c.* 0.832 Ma, 95% CI: 0.485–1.103 Ma) or Middle Pleistocene (Qinghai-Gansu-Inner Mongolia: *c.* 0.370 Ma, 95% CI: 0.172–0.683 Ma) (Table 3).

**TABLE 3:** Results of mismatch distribution analysis (MDA) and neutrality tests for pooled populations of clades of *Scrophularia incisa* complex.

Lineage/subclade	Parameter (τ)	Expansion time (t, Ma)	*SSD*	*P*	*H*_*Rag*_	*P*	Fu’s *Fs*	*P*	Taijima’s *D*	*P*
Xinjiang clade	6.995 (4.080, 9.275)	0.832 (0.485, 1.103)	0.014	0.046	0.033	0.016	2.290	0.800	−0.182	0.536
Tibet clade	0.000 (0.119, 2.027)	NC	0.166	0.014	0.680	0.265	2.628	0.866	1.137	0.851
Qinghai-Gansu-Inner Mongolia clade	3.113 (1.449, 5.740)	0.370 (0.172, 0.683)	0.017	0.428	0.045	0.523	1.697	0.745	−0.079	0.554

Estimates were acquired under a model of demographic expansion using ARLEQUIN. NC, not calculated; Ma, Myr ago.

### Nuclear microsatellite genotyping, population structure, and migration/gene flow

Using FREENA, the frequency of null alleles at each of the 12 nSSR loci was lower than the threshold (ν = 0.15) across the 32 populations of the *S. incisa* complex. There was no evidence for LD and significant deviation from Hardy-Weinberg equilibrium. All populations revealed a high genetic diversity across the 12 loci surveyed, with mean per-locus estimates of allele and gene diversity of *NA* = 16.563 (range: 2–24) and total *HS* = 3.033, along with private gene diversity (*PA*_*R*_ = 0.150), observed heterozygosity (*H*_*O*_ = 0.782) and expected heterozygosity (*H_*E*_* = 0.774) (Supplementary Table 1). At the species level, measures of *H*_*E*_ derived from all 12 loci were highest in *S. incisa* (0.782), followed by *S. kiriloviana* (0.771) and *S. dentata* (0.685). In the hierarchical AMOVA, 8.08% of the total nSSR variation was distributed among the three species (*R*_*CT*_ = 0.08), 11.50% was explained by variation among populations within species (*R*_*SC*_ = 0.13), and 80.41% was apportioned within populations (*R*_*ST*_ = 0.20; Supplementary Table 1). Non-hierarchical analyses revealed less strong population genetic structure in *S. incisa* (*R*_*ST*_ = 0.12), *S. kiriloviana* (*R*_*ST*_ = 0.15), and *S. dentata* (*R*_*ST*_ = 0.03) (Supplementary Table 1).

**FIGURE 4:**
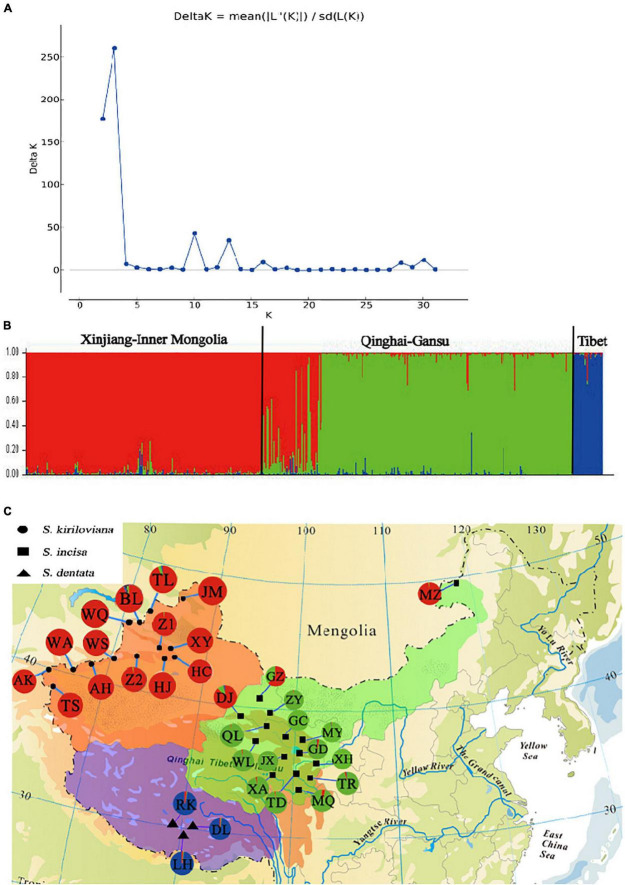
Bayesian clustering results of the STRUCTURE analysis for nSSR data of 530 individuals (32 populations) of *Scrophularia incisa* complex from Northwest China. Number of clusters (*K*) was varied from 1 to 32 in 10 independent runs. **(A)** The dot plot represents changes of the mean posterior probability [log_e_ P(*D*)] (±SD) values of each *K* calculated according to Pritchard et al. (2000), whereas the superimposed line diagram indicates the corresponding Δ*K* statistics calculated according to Evanno et al. (2005). **(B)** Histogram of the STRUCTURE analysis for the model with *K* = 3 (showing the highest Δ*K*). The smallest vertical bar represents one individual. The assignment proportion of each individual into one of three population clusters (or “gene pools”) is shown along the *y*-axis. **(C)** Geographic origin of the 32 *S. incisa* complex populations and their color-coded grouping according to the STRUCTURE analysis. Population codes are identified in Supplementary Table 1.

For the entire nSSR dataset (32 populations, *n* = 522), STRUCTURE yielded the highest likelihood when samples were clustered into three groups (*K* = 3). All individuals of *S. kiriloviana* from Xinjiang and *S. incisa* from Inner Mongolia formed a separate cluster (“red” in Figure 4), whereas those of *S. incisa* from Qinghai and Gansu were assigned to the “green” gene pool; by contrast, all individuals of *S. dentata* from Tibet formed the third (“blue”) cluster. The nSSR dataset thus showed a geographic distribution pattern that was largely congruent with that of cpDNA (Figure 3), apart from a population of *S. incisa* from Inner Mongolia (population MZ), which form a monophyly cpDNA lineage with *S. incisa* from Qinghai-Gansu, but fall into *S. kiriloviana* “red” gene pool according to nSSR result.

Based on the nSSR data, all 12 pairwise estimates of inter-region migration rates were significant (no 95% confidence intervals overlapping zero), ranging from 3.426 (Tibet to Xinjiang) to 10.920 (Xinjiang to Tibet). And it’s also, for instance, the most distinctly obvious asymmetrical pair of migration rates for *S. incisa* complex. There was also relatively high gene flow from Qinghai-Gansu-Inner Mongolia to Tibet (8.9710) and, to a lesser extent, in the reverse direction (4.4978). In general, estimates of inter-region gene flow were of low magnitude and mostly symmetrical (Table 4).

**TABLE 4:** Estimates of migration rate (M) and 95% confidence intervals (CI) (in parentheses) between four *Scrophularia incisa* complex geographic regions using MIGRATE.

Geographic regions	Central Asia	Xinjiang	Tibet	Qinghai-Gansu-Inner Mongolia
Central Asia		6.4145 (6.1487–6.6877)	3.8074 (3.6697–3.9486)	8.8966 (8.6057–9.1941)
Xinjiang	6.6932 (6.4644–6.9275)		**3.4264 (3.2959–3.5604)**	3.9745 (3.7809–4.1746)
Tibet	4.7602 (4.5677–4.9580)	**10.9202 (10.5722–11.2759)**		**8.9710 (8.6788–9.2697)**
Qinghai-Gansu-Inner Mongolia	5.8039 (5.5911–6.0221)	4.8053 (4.5756–5.0424)	**4.4978 (4.3481–4.6513)**	

Asymmetrical gene flow is shown in bold. Directionality of gene flow is read from geographic regions on top being the source populations, whereas geographic units on the left are the recipient populations.

### Predicted distributions and niche identity tests

**FIGURE 5:**
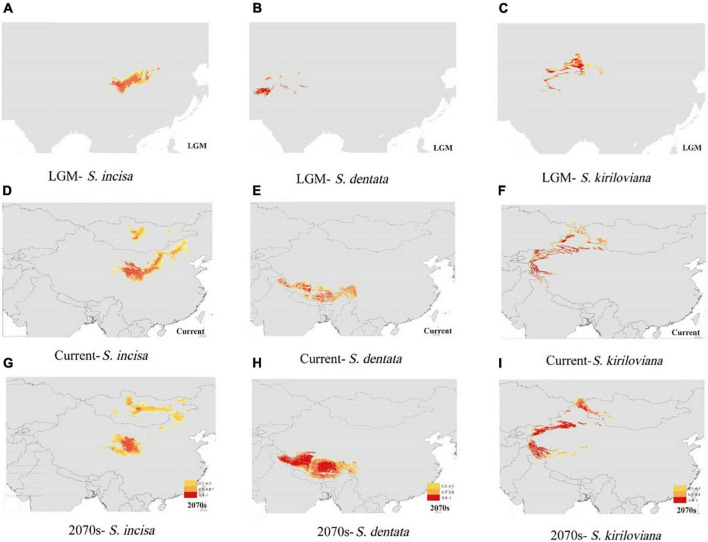
Predicted distributions of S. incisa, S. dentata, and S. kiriloviana at the **(A–C)** Last Glacial Maximum (LGM; 21 000 year before present; BP), under **(D–F)** current conditions (1950–2000), and **(G–I)** future (2070s) based on ecological niche modeling using MAXENT v3.3.1 (Phillips et al., 2006). Maps were generated using ARCGIS v9.3 (ESRI, Redlands, CA, USA).

The constructed ecological niche models performed well for *S. incisa* complex with high AUC scoring above 0.96, indicating good predictive model performance. The most important environmental predictors were precipitation of driest month (BIO14) for *S. dentata*, and precipitation of coldest quarter (BIO19) for *S. incisa* and *S. kiriloviana*. The species’ predicted potential distribution of the species complex under current conditions (1950–2000) was generally similar to their actual distributions (Figure 5). Suitable habitat for *S. incisa* at the LGM contracted greatly in the northeastern part of QTP, whereas *S. dentata* retreated to southern and western parts of the Himalaya Mountains, and *S. kiriloviana* became mainly restricted to the Tianshan Mountains (Figure 5). Suitable habitat for *S. dentata* and *S. kiriloviana* is predicted to become much larger by 2070 with a shift eastwards and expansion northwards and southwards from the Tianshan Mountains, respectively; however, for this future scenario, suitable habitat for *S. incisa* is predicted to become fragmented and extinct in Ningxia and the Midwest of Inner Mongolia (Figure 5).

The results of the niche identity tests undertaken using ENMTools supported the existence of niche differentiation among the three species, the observed values of Schoener’s *D* and Hellinger’s *I* (between *S. incisa* and *S. kiriloviana*: 0.296 and 0.535; between *S. dentata* and *S. incisa*: 0.264 and 0.476; between *S. dentata* and *S. kiriloviana*: 0.267 and 0.503, respectively) were significantly lower than the null distributions, indicating that *S. incisa*, *S. kiriloviana* and *S. dentata* were ecologically distinct species (Supplementary Table 5).

### Ancestral area reconstructions

**FIGURE 6:**
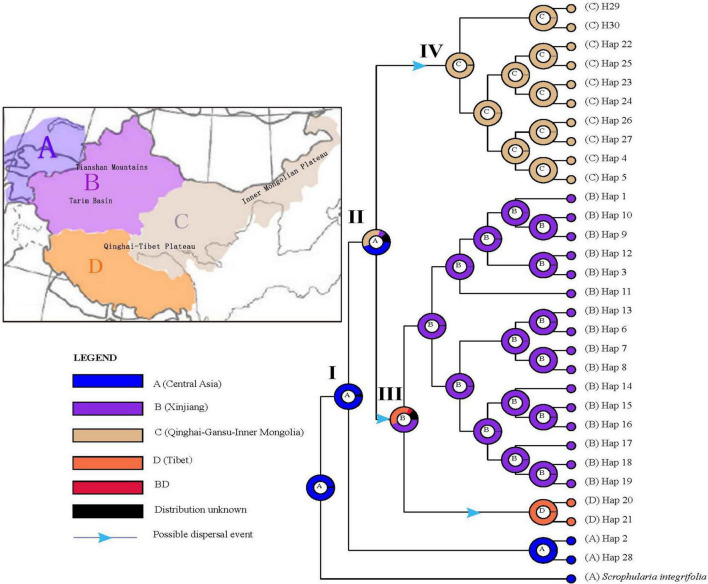
Ancestral area reconstructions based on the Bayesian binary Markov chain Monte Carlo (BBM) method implemented in RASP using the BEAST-derived chronogram of *Scrophularia incisa* complex (see Figure 2). The insert map shows major floristic divisions **(A–D)**. Pie charts of each node illustrate the marginal probabilities for each alternative ancestral area derived from BBM with the maximum area number set to four. The color key identifies possible ancestral ranges at different nodes. Possible dispersal events are indicated by blue arrows.

Based on the topology of the BEAST chronogram (Figure 2), the BBM analysis of ancestral distribution areas (Figure 6) supported a probably ancient (pre-Quaternary) distribution of the *S. incisa* complex in Central Asia (node I). A vicariant event, evident at this node, was likely followed by two independent colonization events from Central Asia (node II) to Xinjiang (B) and Qinghai-Gansu–Inner Mongolia (C), respectively, possibly during the Late Pliocene to Early Pleistocene (see nodes 4, 5, 6 in Figure 2), then followed by a more recent (Pleistocene) dispersal event from Xinjiang (B) to Tibet (D: H20, H21) (see Figure 6); geographical vicariance between these regions was established.

## Discussion

### Species boundaries and cytoplasmic-nuclear discordance in *Scrophularia incisa* complex

Our cpDNA haplotype network (Figure 3) and phylogeny (Figure 2) indicate that the “Central Asia” (CA) clade of *S. kiriloviana* mainly occurs along the eastern foothills of the Pamirs Plateau in Southwest Xinjiang and west of the Tianshan Mountains in Uzbekistan; by contrast, the “China” (CH) clade consists of three subclades in Xinjiang (*S. kiriloviana*), Tibet (*S. dentata*) and Qinghai-Gansu–Inner Mongolia (*S. incisa*), respectively. Based on the nSSR data (Figure 4), the *S. incisa* complex is divided into three gene pools, Xinjiang–Inner Mongolia (*S. kiriloviana* and *S. incisa*), Qinghai-Gansu (*S. incisa*), and Tibet (*S. dentata*), respectively. Hence, this nuclear dataset identifies a similar broad-scale phylogeographic pattern across the complex, as registered by cpDNA, except for a population (MZ) of *S. incisa* from Inner Mongolia fell into the Xinjiang gene pool (Figure 4). Similarly, shared genotypes between the clades were not found in their cpDNA sequence, but appeared in the nSSR datasets. Some individuals in the two populations (GZ and DJ) in northwestern Gansu show dominant red genetic clusters from Xinjiang gene pool and the rest populations from Qinghai-Gansu show little red genetic cluster from Xinjiang gene pool and blue genetic cluster from Tibet (Figure 4). The same situation to populations form Xinjiang and Tibet clade that have more or less genetic clusters from other gene pools, which maybe due to the mostly low magnitude of symmetrical inter-region gene flow (Table 4). We suggest that there are three recognized species, and several examples of incongruence between the cpDNA and nSSR data sets indicate that introgression had occurred among the three species, and/or lineage sorting between different groups was incomplete (Wang et al., 2008; Lin et al., 2019).

### Allopatric divergence and evolution history

Our cpDNA results show that the *S. incisa* complex is comprised of two major clades [Central Asia (CA) vs. China], with unique sets of haplotypes and distinct geographic distributions (Figure 3). In conjunction with BEAST-derived our molecular dating (Figure 2), the ancestral range reconstructions (Figure 6) indicate that the split of CA and China clades during the Middle Pliocene at *c*. 3.36 (2.12–4.68) Ma. Subsequently, the China clade is further divided into four subclades, namely, “Xinjiang” (*c*. 2.12), “Tibet” (*c*. 0.73), “Qinghai-Gansu” (*c*. 1.17), and “Inner Mongolia” (*c*. 0.72), respectively (Figure 2). Most studies support species divergences in the last 5 Ma, such as in the genera of *Rheum* L., *Sinacalia* H. Rob. and Brettell and *Solms-laubachia* (Maxim.) Botsch (Wang et al., 2005; Liu et al., 2006; Yue et al., 2009), except some plant lineages diverged following the early uplifts of the QTP, or even prior to the formation of the plateau (Liu et al., 2002; Yang et al., 2003; Zhang and Fritsch, 2010; Sun et al., 2012). However, extensive heterogeneous uplift events across the QTP *sensu lato* (*sl*) which have occurred from the Miocene to Pliocene according to available evidence (Manish and Pandit, 2018; Fang et al., 2020; Mao et al., 2021), in association with a monsoon-dominated climate pattern (An et al., 2001; Guo et al., 2008; Spicer, 2017) and global climate cooling (Shackleton et al., 1995; Zachos et al., 2001) triggered aridification increasement and sand deserts enlargement in Northwestern China (Sun et al., 2008; Miao et al., 2012). These climatic and geological changes may have intensified the vicariance and fragmentation, which played a key role in plant morphogenesis and adaptive evolution (Xu et al., 2022), further facilitated the plant allopatric speciation and divergence into different lineages and species (Qiu et al., 2011; Li et al., 2012; Wen et al., 2016). This inference of eco-geographically driven, and possible adaptive divergence is further illustrated by our niche modeling, which indicates that the three species occupy significantly different climatic environments (Supplementary Table 5).

Underlying reasons of how organisms are spread geographically is a core objective of biogeography (Cox and Moore, 2000). Reviewing recent evidences from phylogenetic and biogeographic studies in plants, the Tethyan Tertiary flora and the Arcto-Tertiary flora have been indicated as important sources of the QTP alpine and forest floras (Sun, 2002; Liao et al., 2012; Zhou et al., 2013; Wen et al., 2014). Some others suggested that majority of these temperate groups originated on the QTP and subsequently migrated into the other regions of Eurasia, during global temperature decreased and the QTP uplifted extensively, i.e., the out-of-QTP hypothesis (Ozenda, 1988; Jia et al., 2012; Fan et al., 2013; Zhang J. Q. et al., 2014). CpDNA haplotype variation phylogenetic tree demonstrated that the basal phylogenetically clades of the *S. incisa* complex were exclusively distributed in the Central Asia (Figure 2), supporting the hypothesis of “migrations/dispersals from Central Asia into the QTP,” as inferred from various genera, such as *Solms-laubachia*, *Incarvillea*, and *Myricaria* (Grierson, 1961; Chen et al., 2005; Wang et al., 2009; Yue et al., 2009). Further evidence of this hypothesis came from RASP-BBM analysis of the cpDNA tree (Figure 6), which suggested that Central Asia, cradle of the *S. incisa* complex and multiple dispersals from Central Asia accounted for most of the species’ range expansion in Asia. The inferred migration route of *S. incisa* complex to China through intervening mountain ranges in northwestern Himalaya is one suggested previously for many plants of the QTP and adjacent regions that originated in the Central Asia region (Yue et al., 2009). Another corridor from Central Asia *via* the north of Xinjiang to Mongolia Plateau, Qinghai and Gansu is also reported before (Chen et al., 2005; Jia et al., 2012). It was estimated that two dispersal routes to QTP *via* northwestern Himalaya to Tibet and *via* northern Xinjiang to Inner Mongolia, Qinghai and Gansu with both dispersal events having their source in Central Asia. The phylogeographic patterns based on the maternally inherited cpDNA are likely to reflect past migration of the species through long-distance seed dispersal mediated by river. According to our field investigation, the *S. incisa* complex is usually distributed along the alpine water of the melt snow in the valley, floodplains, and streams, we speculate the seeds in capsule propagate by river.

### Glacial refugia, habitat fragmentation, and conservation implications

Among the remarkable climatic changes of the Cenozoic, the climatic oscillations during the Quaternary had great influence on the genetic structure and distribution of extant plants in China (Comes and Kadereit, 1998; Hewitt, 2000; Wen et al., 2016). Species experienced glacial-time retreat to separate refugia and interglacial recolonization in response to cold-warm climatic cycles (Hewitt, 2000), separate refugia are hypothesized to result in fragmentation of the geographical distributions, deep allopatric divergence and intraspecific differentiation, especially the plants in the Altay-Tianshan Mountains (Tang et al., 2010; Wu et al., 2010; Meng et al., 2015). Our genetic and ENM evidence for S. *kiriloviana* in Xinjiang and *S. incisa* in Qinghai-Gansu have shown significant phylogeographical structure with the location of refugia in the Altay-Tianshan Mountains and southeast of Qinghai Plateau, as considered the biotic glacial refugium where plants persisted during glacial periods (Meng et al., 2015). The humid valleys from Altay-Tianshan Mountains may provide refugial habitats for *S. kiriloviana*, while the distribution of most our sampled populations of *S. kiriloviana* from Xinjiang were fragmented in different valleys. *S. incisa* in Qinghai-Gansu occurring on the plateau platform was speculated to experience postglacial expansion and recolonization from the refugia located at lower elevations at the southeast plateau edge (Li et al., 2011; Qiu et al., 2011), although the expansion signal pertaining to the Qinghai-Gansu-Inner Mongolia clade most likely occurred before the LGM and cannot be linked to a major postglacial demographic event, which maybe due to the influence of population from Inner Mongolia on the demographic analysis and expansion time estimation of Qinghai-Gansu populations. Moreover, as our ENM results predict fragmented distribution for *S. incisa* and extinction in Ningxia and midwestern Inner Mongolia by 2070 (Figure 5), which is consistent with our field investigations that its current population number and size appears limited in midwestern Inner Mongolia, possibly as a consequence of over-exploitation and habitat destruction by human activity for its medicinal value (Guo et al., 2019; Han et al., 2022), and none individuals were collected during our fieldwork. So we suggest that *in situ* conservation efforts should be made such as establishing protected areas and population recovering in natural habitat for *S. incisa* (Zhou et al., 2021).

## Conclusion

Our phylogeographic and niche modeling evidences suggest that there are three genetically and ecological recognized species, comparison of the topologies of cpDNA network/tree and nSSR structure revealed several examples of incongruence indication that historical hybridization or incomplete lineage sorting had occurred among the three species and between different clades. The *S. incisa* complex diverged at the molecular level into several distinct clades, as resolved in the cpDNA phylogenetic tree and network. Our molecular dating revealed that the origin of these clades dates back to the Middle Pliocene, *c.* 3.94 Ma, when the QTP and other parts of Eurasia underwent considerable geological change and/or climatic oscillations. Such paleo-events are likely to have fragmented the distribution of the complex and triggered allopatric divergence and the formation of deep clades. Our phylogenetic based AAR evidences support that *S. incisa* complex originated in the Central Asia with subsequent dispersals from the Central Asia into Northwestern China and diversification of several groups in the Tianshan Mountains, the QTP and the Inner Mongolian Plateau. This work therefore provides a further example of the overriding role of the Plio-Pleistocene climate change, in this case aridification triggering allopatric speciation and diversification in this group of steppe and desert plants of Northwestern China.

## Data availability statement

The cpDNA sequences data obtained in this study are deposited in GenBank (see Supplementary Table 2 for accession number). Microsatellite genotypes and maxent input file are openly available in Github at https://github.com/ruihongwang77/Microsatellite-genotypes-and-maxent-data-of-Scrophularia-incisa-complex.

## Author contributions

C-XF conceived and designed the study. PL, C-XF, R-HW, and Z-PY collected the samples. R-HW performed the experiments, analyzed the data, and wrote drafts of the manuscript. Z-CZ participated in the data analysis. HC and Z-CQ helped to improve the final manuscript. All authors contributed to the article and approved the submitted version.

## Footnotes

1
www.cvh.ac.cn

2
www.nsii.org.cn

3
www.gbif.org

4
http://www.worldclim.org

5
http://mnh.scu.edu.cn/soft/blog/RASP
