# Supplementary material for: Plio-Pleistocene climatic change drives allopatric speciation and population divergence within the Scrophularia incisa complex (Scrophulariaceae) of desert and steppe subshrubs in Northwest China
Source: Front Plant Sci. 2022 Sep 21;13:985372. doi: 10.3389/fpls.2022.985372 (PMC9532938; doi:10.3389/fpls.2022.985372)
Supplement: Supplementary file 1 [file Data_Sheet_1.PDF]

Supplementary Material

Supplementary Figures

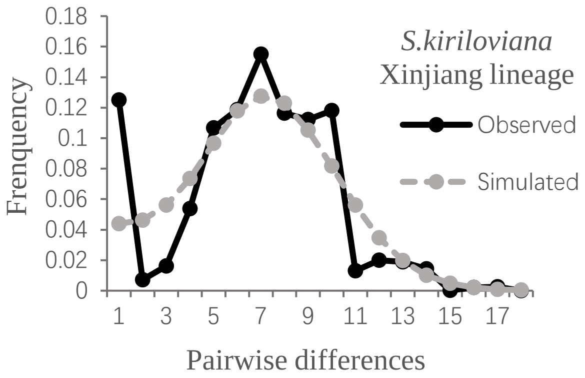

(a)

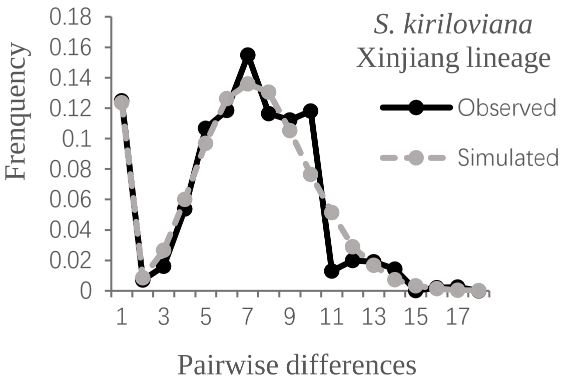

(b)

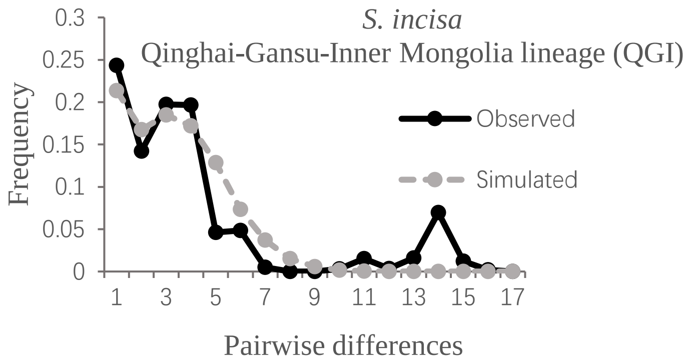

(c)

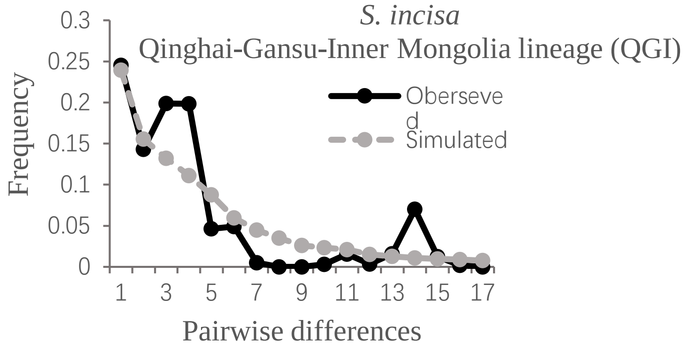

(d)

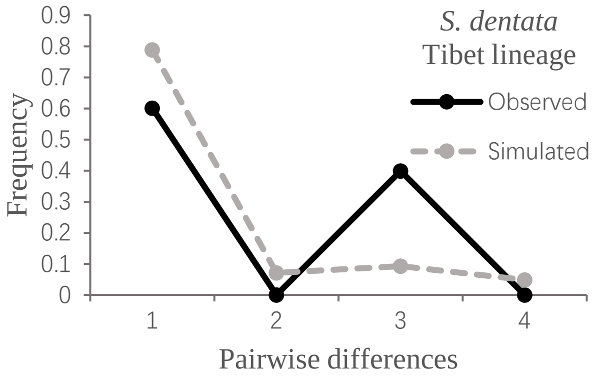

(e)

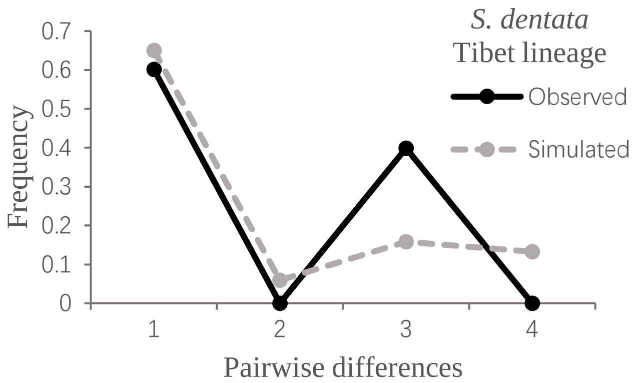

(f)

Figure S1. Mismatch distribution analyses (MDAs) of the three cpDNA lineages of the *S.incisa* complex (Xinjiang lineage: *S. kiriloviana*; Qinghai-Gansu-Inner Mongolia lineage: *S. incisa*; Tibet lineage: *S. dentata*) for the pure demographic expansion model (a, c, and e) and the spatial expansion model (b, d, and f).

Supplementary Tables

Table S1. Geographic and genetic characteristics of *Scrophularia incisa* complex population samples used in this study. *n*, number of individuals; *h*, haplotype diversity; π, nucleotide diversity; A_R, allelic richness; P_{AR}, private allelic richness; H_E, expected heterozygosity; H_O, observed heterozygosity; F_{IS}, fixation index.

Species/ Population Code	Locations	Latitude (°N)	Longitude (°E)	Altitude (m)	N (cpDNA/ nSSR)	cpDNA			nSSRs				
						h (±SD)	$\pi \times 10^{-3}$ (±SD)	haplotypes (no. of individuals)	A_R	PA_R	H_E	H_O	F_{IS}
S. kiriloviana (15 populations)													
AK	Aketao, Xinjiang	38.76	75.19	2790	15/20	0.000	0.000	H2(15)	2.79	0.16	0.821	0.829	0.015
TS	Jimu,Tashi,Xinjiang	38.74	75.04	3286	15/21	0.000	0.000	H2(15)	3.11	0.16	0.841	0.782	0.207
CA	West Tien-Shan, Chofkae Rande Raffasai, Uzbekistan	41.15	70.13	1656	3/0	0.000	0.000	H28(3)					
WA	Wuqia, Xinjiang	39.80	74.80	2752	13/24	0.248	2.160	H2(2), H8(11)	3.14	0.15	0.842	0.757	0.122
AH	Aheqi, Xinjiang	40.75	77.83	2761	15/24	0.000	0.000	H1(15)	3.20	0.11	0.813	0.757	0.091
WS	Wensu, Xinjiang	41.79	80.67	2235	10/11	0.000	0.000	H1(14)	2.72	0.26	0.793	0.803	0.035
Z1	Zhaosu, Xinjiang	43.20	81.20	1952	12/13	0.773	1.600	H7(1),H14(3),H15(4), H16(4)	2.94	0.15	0.816	0.825	-0.004
Z2	Zhaosu, Xinjiang	43.19	80.96	2085	13/16	0.538	0.440	H17(6), H18(3), H19(4)	3.14	0.12	0.660	0.750	0.05
XY	Xinyuan, Xinjiang	43.25	84.30	1725	15/20	0.133	0.100	H7(1), H13(14)	3.28	0.14	0.816	0.825	0.015
HC	Huocheng, Xinjiang	44.42	81.04	1408	5/8	0.286	0.220	H6(1), H7(4)	3.25	0.49	0.664	0.646	-0.029
HJ	Hejing, Xinjiang	42.91	86.19		13/18	0.000	0.000	H8(13)	3.29	0.09	0.831	0.880	0.095
WQ	Wenquan, Xinjiang	45.08	81.16	1446	5/5	0.000	0.000	H12(5)	3.18	0.06	0.660	0.750	-0.026
BL	Bole, Xinjiang	45.17	82.11	1478	4/4	0.000	0.000	H3(4)	2.73	0.01	0.648	0.729	0.094
TL	Tuoli, Xinjiang	45.79	83.61	1653	14/20	0.000	0.000	H10(14)	3.27	0.26	0.799	0.629	0.122
JM	Jimunai, Xinjiang	47.11	86.63		14/16	0.000	0.000	H9(14)	3.12	0.08	0.796	0.656	0.237
	**Species mean**					**0.132 (0.011)**	**0.302 (0.130)**		**3.083**	**0.16**	**0.771**	**0.758**	
S. incisa (15 populations)													
DJ	Dangjin, Gansu	39.12	93.40		15/17	0.000	0.000	H5(15)	2.90	0.10	0.810	0.725	0.136
GC	Gangca, Qinghai	37.14	100.34	3201	15/20	0.000	0.000	H22(15)	3.17	0.07	0.747	0.704	0.083
GZ	Guazhou, Gansu	39.95	96.49		15/19	0.419	0.330	H4(4), H5(11)	2.81	0.11	0.835	0.807	0.061
GD	Gandi, Qinghai	36.34	100.43	3125	14/20	0.000	0.000	H22(14)	3.26	0.10	0.814	0.846	-0.013
JX	Jiangxigou, Qinghai	36.58	100.50	3200	15/20	0.000	0.000	H22(15)	3.19	0.08	0.724	0.742	0.001
MQ	Maqin, Qinghai	34.68	100.69	3275	15/20	0.000	0.000	H23(14)	3.06	0.19	0.821	0.825	0.021

MY	Menyuan, Qinghai	37.11	102.35	2452	15/20	0.000	0.000	H24(15)	2.96	0.15	0.789	0.813	-0.005
QL	Qilian, Qinghai	38.17	100.01	2985	15/20	0.000	0.000	H22(15)	2.71	0.23	0.760	0.763	0.023
TD	Tongde, Qinghai	35.25	100.44	3032	12/12	0.000	0.000	H22(12)	3.16	0.18	0.677	0.646	0.09
TR	Tongren, Qinghai	35.57	102.19	2708	15/20	0.000	0.000	H25(15)	3.02	0.18	0.814	0.883	-0.06
WL	Wulan, Qinghai	37.01	98.68	3235	14/20	0.000	0.000	H22(14)	3.09	0.11	0.779	0.779	0.026
XH	Xunhua, Qinghai	35.83	102.64	1888	15/17	0.000	0.000	H22(14)	3.09	0.15	0.786	0.863	-0.073
ZY	Zhangye, Gansu	38.54	100.25	2753	15/20	0.476	0.370	H26(10), H27(5)	3.16	0.06	0.796	0.759	0.074
XA	Xinghai, Qinghai	36.08	99.85	3323	15/18	0.000	0.000	H22(15)	3.01	0.08	0.791	0.824	-0.011
MZ	Manzhouli, Inner Mongolia	41.79	80.67	2191	14/20	0.000	0.000	H29(10),H30(4)	3.14	0.16	0.809	0.842	-0.015
	**Species mean**					**0.060 (0.041)**	**0.047 (0.040)**		**3.042**	**0.128**	**0.782**	**0.784**	
S. dentata (3 populations)													
DL	Duilong, Tibet				3/2	0.000	0.000	H20(3)	2.86	0.12	0.573	0.917	-0.333
LH	Linze, Tibet				9/10	0.389	0.310	H20(2), H21(7)	2.75	0.10	0.723	0.767	-0.008
RK	Rikaze, Tibet				14/15	0.000	0.000	H20(14)	3.48	0.23	0.760	0.822	-0.048
	**Species mean**					**0.130 (0.084)**	**0.103 (0.070)**		**3.03**	**0.15**	**0.685**	**0.835**	
**Total mean**						**0.102 (0.017)**	**0.314 (0.100)**				**0.774 (0.006)**	**0.782 (0.009)**	

Table S2. GenBank accession numbers of all newly generated cpDNA sequences of *Scrophularia incisa* complex.

	Genebank accession number		
Haplotype	*psb*A-*trn*H	*trn*L-*trn*F	*trn*Q-*rps*16
H1	MW657243	MW657273	MW657303
H2	MW657244	MW657274	MW657304
H3	MW657246	MW657275	MW657305
H4	MW657245	MW657276	MW657306
H5	MW657247	MW657277	MW657307
H6	MW657248	MW657278	MW657308
H7	MW657249	MW657279	MW657309
H8	MW657250	MW657280	MW657310
H9	MW657251	MW657281	MW657311
H10	MW657252	MW657282	MW657312
H11	MW657253	MW657283	MW657313
H12	MW657254	MW657284	MW657314
H13	MW657255	MW657285	MW657315
H14	MW657256	MW657286	MW657316
H15	MW657257	MW657287	MW657317
H16	MW657258	MW657288	MW657318
H17	MW657259	MW657289	MW657319
H18	MW657260	MW657290	MW657320
H19	MW657261	MW657291	MW657321
H20	MW657262	MW657292	MW657322
H21	MW657263	MW657293	MW657323
H22	MW657264	MW657294	MW657324
H23	MW657265	MW657295	MW657325
H24	MW657266	MW657296	MW657326
H25	MW657267	MW657297	MW657327
H26	MW657268	MW657298	MW657328
H27	MW657269	MW657299	MW657329
H28	MW657270	MW657300	MW657330
H29	MW657271	MW657301	MW657331
H30	MW657272	MW657302	MW657332

Table S3. Characteristics of the 12 microsatellite loci surveyed across *Scrophularia incisa* populations

Locus	Size range (bp)	N_A	H_O	H_E	H_S	H_T	F_{ST}	G'_{ST}
Scin1	109-128	26	0.760	0.910	0.789	0.917	0.143	0.140
Scin2	140-158	18	0.783	0.839	0.747	0.851	0.102	0.122
Scin3	144-162	31	0.599	0.835	0.720	0.838	0.127	0.140
Scin4	225-243	28	0.783	0.942	0.842	0.944	0.090	0.107
Scin5	291-309	25	0.758	0.882	0.767	0.880	0.129	0.128
Scin6	113-132	28	0.888	0.930	0.879	0.926	0.048	0.051
Scin7	183-201	38	0.718	0.955	0.873	0.954	0.074	0.085
Scin8	107-126	24	0.873	0.915	0.869	0.920	0.046	0.055
Scin9	110-128	28	0.845	0.906	0.816	0.911	0.103	0.104
Scin10	144-164	30	0.819	0.932	0.813	0.934	0.115	0.129
Scin11	273-294	24	0.749	0.890	0.783	0.890	0.112	0.121
Scin12	132-162	28	0.836	0.922	0.814	0.921	0.113	0.117
Mean	——	27	0.784	0.905	0.809	0.907	0.099	0.108

N_A, number of alleles per locus; H_O, observed heterozygosity; H_E, expected heterozygosity; H_S, expected genetic diversity within populations; H_T, overall gene diversity; F_{ST}, among population differentiation (Weir & Cockerham, 1984); G'_{ST} standardized measure of genetic differentiation (Hedrick, 2005).

Table S4 Chloroplast DNA sequences polymorphisms detected in the *psb*A-*trn*H, *trn*L-*trn*F and *trn*Q-*rps*16 in *S. incisa* complex.

Haplotype	Nucleotide position																					
	*psb*A-*trn*H																					
	8	17	54	63	76	79	81	97	155	179	202	204	207	234	240	259	273	286	321	328	359	380	387
H1	T	A	G	---	C	T	C	1	A	C	C	A	1[a]	A	C	C	T	T	ATATC	1[b]	G	TAA	GACT
H2	C	G	T	---	.	.	.	2	.	.	.	.	-	.	.	.	.	.	ATATC	1[b]	.	TAA	GACT
H3	.	.	.	---	.	.	.	1	.	T	.	.	1[a]	G	.	.	.	.	ATATC	1[b]	.	TAA	GACT
H4	.	.	.	TAA	.	.	.	2	.	.	G	-	1[a]	.	T	.	.	C	-----	1[b]	T	TAA	----
H5	.	.	.	TAA	.	.	.	2	.	.	G	-	1[a]	.	T	.	.	C	-----	1[b]	T	TAA	----
H6	.	.	.	---	.	.	.	2	C	.	.	.	1[a]	.	T	.	G	.	ATATC	1[b]	T	TAA	GACT
H7	.	.	.	---	.	.	.	1	C	.	.	.	1[a]	.	T	.	G	.	ATATC	1[b]	T	TAA	GACT
H8	.	.	.	---	.	.	.	1	C	.	.	.	1[a]	.	T	.	G	.	ATATC	1[b]	T	TAA	GACT
H9	.	.	.	---	.	.	.	1	.	.	.	.	1[a]	.	T	.	.	.	ATATC	1[b]	.	TAA	GACT
H10	.	.	.	---	.	.	.	1	.	.	.	.	1[a]	.	T	.	.	.	ATATC	1[b]	.	TAA	GACT
H11	.	.	.	---	T	.	T	1	.	.	.	.	1[a]	.	T	.	.	.	ATATC	1[b]	.	TAA	GACT
H12	.	.	.	---	.	.	.	1	.	.	.	.	1[a]	.	T	T	.	.	ATATC	1[b]	.	TAA	GACT
H13	.	.	.	---	.	.	.	2	C	.	.	.	1[a]	.	T	.	G	.	ATATC	1[b]	T	TAA	GACT
H14	.	.	.	---	.	.	.	1	.	.	.	.	1[a]	.	T	.	.	.	ATATC	1[b]	.	TAA	GACT
H15	.	.	.	---	.	A	.	1	.	.	.	.	1[a]	.	T	.	.	.	ATATC	1[b]	.	TAA	GACT
H16	.	.	.	---	.	A	.	2	.	.	.	.	1[a]	.	T	.	.	.	ATATC	1[b]	.	TAA	GACT
H17	.	.	.	---	.	.	.	1	.	.	.	.	1[a]	.	T	.	.	.	ATATC	-	.	TAA	GACT
H18	.	.	.	---	.	.	.	2	.	.	.	.	1[a]	.	T	.	.	.	ATATC	-	.	TAA	GACT
H19	.	.	.	---	.	.	.	2	.	.	.	.	1[a]	.	T	.	.	.	ATATC	-	.	TAA	GACT
H20	.	.	.	---	.	.	.	1	.	.	.	.	1[a]	.	T	.	.	.	ATATC	1[b]	.	TAA	GACT
H21	.	.	.	---	.	.	.	2	.	.	.	.	1[a]	.	T	.	.	.	ATATC	1[b]	.	TAA	GACT
H22	.	.	.	TAA	.	.	.	2	.	.	G	-	1[a]	.	.	.	.	C	ATATC	1[b]	T	TAA	GACT
H23	.	.	.	TAA	.	.	.	2	.	.	G	-	1[a]	.	.	.	.	C	ATATC	1[b]	T	TAA	GACT
H24	.	.	.	TAA	.	.	.	2	.	.	G	-	1[a]	.	.	.	.	C	ATATC	1[b]	T	---	GACT
H25	.	.	.	TAA	.	.	.	2	.	.	G	-	1[a]	.	.	.	.	C	ATATC	1[C]	A	TAA	GACT
H26	.	.	.	TAA	.	.	.	1	.	.	G	-	1[a]	.	.	.	.	C	ATATC	1[b]	T	TAA	----
H27	.	.	.	TAA	.	.	.	2	.	.	G	-	1[a]	.	.	.	.	C	ATATC	1[b]	T	TAA	----
H28	C	.	T	---	.	.		2	.	.	.	.	1[a]	.	.	.	.	.	ATATC	1[b]	.	TAA	GACT
H29	.	.	.	TAA	.	.		2	.	.	.	-	1[a]	.	.	.	.	.	ATATC	1[b]	.	TAA	GACT
H30	.	.	.	TAA	.	.		2	.	.	.	-	1[a]	.	.	.	.	.	ATATC	1[b]	.	TAA	GACT

a,AAAGAAGA;b,AAAAATAAGAAAGAAGATAAATGAAATGATTGAAATTCTATTTTTGTG;c,AAAAAAAAGAAAGAAGATAAATGAAATGATTGAAATTCTATTTTTGTG;
1,TTTTTCTTCTTCTATCAAGAGG;2,CCTCTTGATAGAAGAAGAAAAA
AAAAATAAGAAAGAAGATAAATGAAATGATTGAAATTCTATTTTTGTG
AAAAAAAAGAAAGAAGATAAATGAAATGATTGAAATTCTATTTTTGTG

Haplotype	Nucleotide position																	
	*trn*L-*trn*F																	
	71	202	228	255	269	289	339	381	404	545	546	548	561	634	647	665	679	783
H1	T	A	-	G	A	G	2	-	A	A	G	T	T	C	T	C	A	G
H2	.	G	-	.	C	.	2	-	G	.	T	.	.	.	.	.	.	.
H3	C	.	-	.	.	.	2	-	G	.	T	.	.	.	.	.	.	.
H4	.	.	-	A	.	.	2	T	G	.	T	.	.	A	.	.	.	.
H5	.	.	-	A	.	.	2	T	G	.	T	.	.	A	.	.	.	.
H6	.	.	-	.	.	.	-	-	G	.	T	.	.	.	.	A	.	.
H7	.	.	-	.	.	.	2	-.	G	.	T	.	.	.	.	A	.	.
H8	.	.	-	.	.	.	2	-	G	.	T	G	.	.	.	A	.	.
H9	.	.	-	.	.	.	2	-	G	.	T	C	.	.	.	.	.	.
H10	.	.	-	.	.	.	2	-	G	.	T	.	C	.	.	.	.	.
H11	.	.	-	.	.	.	2	-	G	C	T	.	.	.	G	.	.	A
H12	.	.	-	.	.	.	2	-	G	.	T	.	.	.	.	.	.	.
H13	.	.	-	.	.	.	2	-	G	.	T	.	.	.	.	A	.	.
H14	.	.	-	A	.	C	2	-	G	.	T	.	.	.	.	A	.	.
H15	.	.	-	.	.	C	2	-	G	.	T	.	.	.	.	A	.	.
H16	.	.	-	.	.	C	2	-	G	.	T	.	.	.	.	A	.	.
H17	.	.	-	.	.	C	2	-	G	.	T	.	.	.	.	A	.	.
H18	.	.	-	.	.	C	2	-	G	.	T	.	.	.	.	A	.	.
H19	.	.	-	.	.	C	2	-	G	.	T	.	.	.	.	.	.	.
H20	.	.	-	.	.	.	2	-	G	.	T	.	.	.	.	.	T	.
H21	.	.	-	.	.	.	2	-	G	.	T	.	.	.	.	.	T	.
H22	.	.	-	A	.	.	2	T	G	.	T	.	.	A	.	.	.	.
H23	.	.	1	A	.	.	2	-	G	.	T	.	.	A	.	.	.	.
H24	.	.	-	A	.	.	2	-	G	.	T	.	.	A	.	.	.	.
H25	.	.	-	A	.	.	2	T	G	.	T	.	.	A	.	.	.	.
H26	.	.	-	A	.	.	2	T	G	.	T	.	.	A	.	.	.	.
H27	.	.	-	A	.	.	2	T	G	.	T	.	.	A	.	.	.	.
H28	.	G	-	.	.	.	2	-	G	.	.	.	.	.	.	.	.	.
H29	.	.	-	.	.	.	2	-	G	.	.	.	.	.	.	A	.	.
H30	.	.	-	A	.	.	2	-	G	.	.	.	.	A	.	.	.	.

1,TCAAAAT; 2, ATTC

Haplotype	Nucleotide position																													
	*trn*Q-*rps*16																													
	70	93	193	246	283	298	312	349	352	358	365	372	381	385	441	459	477	478	519	586	587	597	660	662	669	721	746	756	799	832
H1	C	G	T	T	G	C	T	A	T	C	C	C	A	T	T	T	G	A	T	G	G	A	T	G	T	T	G	C	C	G
H2	.	.	.	.	.	T	G	.	.	.	.	.	.	G	C	.	A	G	.	.	.	.	.	T	.	C	.	.	T	.
H3	.	.	.	.	.	.	.	.	C	.	.	.	.	.	.	.	.	.	.	.	.	.	.	T	C	.	.	.	T	.
H4	.	.	.	.	.	.	.	G	.	.	.	.	T	.	.	.	.	.	.	.	A	.	.	T	T	.	.	.	T	.
H5	.	.	.	.	.	.	.	.	.	.	.	.	.	.	.	.	.	.	.	.	.	.	.	T	T	.	.	.	T	.
H6	.	T	.	.	.	.	.	.	.	.	.	.	.	.	.	.	.	.	.	.	.	.	.	T	T	.	.	.	T	.
H7	.	.	.	.	.	.	.	.	.	.	.	.	.	.	.	.	.	.	.	.	.	.	.	T	T	.	.	.	T	.
H8	.	.	.	.	.	.	.	.	.	.	.	.	.	.	.	.	.	.	.	.	.	.	.	T	T	.	.	.	T	.
H9	.	.	.	.	.	.	.	.	.	.	.	T	.	.	.	.	.	.	.	.	.	.	.	T	T	.	.	.	T	.
H10	.	.	.	.	.	.	.	.	.	.	.	.	.	.	.	.	.	.	G	.	.	.	.	T	T	.	.	.	T	.
H11	.	.	G	.	.	.	.	.	.	T	.	.	.	.	.	.	.	.	.	.	.	.	C	T	T	.	.	T	T	.
H12	.	.	.	.	.	.	.	.	.	.	A	.	.	.	.	C	.	.	.	.	.	.	.	T	T	.	.	.	T	.
H13	.	.	.	.	.	.	.	.	.	.	.	.	.	.	.	.	.	.	.	.	.	.	.	T	T	.	.	.	T	.
H14	.	.	.	.	A	.	.	.	.	.	.	.	.	.	.	.	.	.	.	.	.	.	.	T	T	.	.	.	T	.
H15	.	.	.	.	A	.	.	.	.	.	.	.	.	.	.	.	.	.	.	.	.	.	.	T	T	.	.	.	T	.
H16	.	.	.	.	A	.	.	.	.	.	.	.	.	.	.	.	.	.	.	.	.	.	.	T	T	.	.	.	T	.
H17	.	.	.	.	A	.	.	.	.	.	.	.	.	.	.	.	.	.	.	.	.	.	.	T	T	.	.	.	T	.
H18	.	.	.	.	A	.	.	.	.	.	.	.	.	.	.	.	.	.	.	.	.	.	.	T	T	.	.	.	T	.
H19	.	.	.	.	A	.	.	.	.	.	.	.	.	.	.	.	.	.	.	.	.	.	.	T	T	.	.	.	T	.
H20	.	.	.	.	.	.	.	.	.	.	.	.	.	.	.	.	.	.	.	T	.	.	.	T	T	.	.	.	T	T
H21	.	.	.	.	.	.	.	.	.	.	.	.	.	.	.	.	.	.	.	T	.	T	.	T	T	.	T	.	T	T
H22	.	.	.	G	.	.	.	G	.	.	.	.	T	.	.	.	.	.	.	.	A	T	.	T	T	.	.	.	T	.
H23	.	.	.	.	.	.	.	G	.	.	.	.	T	.	.	.	.	.	.	.	A	.	.	T	T	.	.	.	T	.
H24	.	.	.	.	.	.	.	G	.	.	.	.	T	.	.	.	.	.	.	.	A	.	.	T	T	.	.	.	T	.
H25	.	.	.	.	.	.	.	G	.	.	.	.	T	.	.	.	.	.	.	.	A	.	.	T	T	.	.	.	T	.
H26	.	.	.	.	.	.	.	G	.	.	.	.	T	.	.	.	.	.	.	.	A	.	.	T	T	.	.	.	T	.
H27	.	.	.	.	.	.	.	G	.	.	.	.	T	.	.	.	.	.	.	.	A	.	.	T	T	.	.	.	T	.
H28	T	.	.	.	.	T	G	.	.	.	.	.	.	.	C	.	A	T	.	.	.	.	.	T	T	.	.	.	T	.
H29	.	.	.	.	.	.	.	.	.	.	.	-	.	.	.	.	.	.	.	.	A	.	.	T	T	.	.	.	T	.
H30	.	.	.	.	.	.	.	.	.	.	.	-	.	.	.	.	.	.	.	.	A	.	.	T	T	.	.	.	T	.

Table S5 The results of identity tests among *S. incisa* (SC), *S. kiriloviana* (SK) and *S. dentata* (SD), respectively.

Species paired	Statistical test value	*Schoener's D*	*Hellinger's I*
SC and *SK*	Empirical value	0.296	0.535
	Permuted critical value	0.658	0.890
SD and *SC*	Empirical value	0.264	0.476
	Permuted critical value	0.643	0.879
SD and *SK*	Empirical value	0.267	0.503
	Permuted critical value	0.649	0.880
